# The Increased Type-1 and Type-2 Chemokine Levels in Children with Acute RSV Infection Alter the Development of Adaptive Immune Responses

**DOI:** 10.1155/2014/750521

**Published:** 2014-06-11

**Authors:** Valerija Vojvoda, Ana Savić Mlakar, Mladen Jergović, Mirela Kukuruzović, Leo Markovinović, Neda Aberle, Sabina Rabatić, Krešo Bendelja

**Affiliations:** ^1^Center for Research and Knowledge Transfer in Biotechnology, University of Zagreb, Rockefellerova 10, 10000 Zagreb, Croatia; ^2^Faculty of Food Technology and Biotechnology, University of Zagreb, Pierotti Street 6, 10000 Zagreb, Croatia; ^3^University Hospital for Infectious Diseases “Dr. Fran Mihaljević”, Mirogojska 8, 10000 Zagreb, Croatia; ^4^Department of Pediatrics, General Hospital “Dr. Josip Benčević”, Andrije Štampara 42, 35000 Slavonski Brod, Croatia; ^5^Institute of Immunology, Rockefellerova 2, 10000 Zagreb, Croatia; ^6^National Jewish Health, 1400 Jackson Street, Denver, CO 80206, USA

## Abstract

Severe RSV infections and frequent recurrence could be related to the altered polarization of type-2/type-1 T cells. This increases the importance of determining distinctive chemokines and chemokine receptor profiles on memory T cells. We analyzed systemic adaptive T cell response in the acute (*n* = 17) and convalescent phase (*n* = 7) of RSV-infected children, in the acute (*n* = 11) and convalescent phase (*n* = 6) of children with other viral respiratory infections (adenovirus and influenza virus), and in healthy children (*n* = 18). Expression of CCR4 and CXCR3 on effector-memory (T_EM_) and central-memory (T_CM_) T cells was compared between tested groups. Serum concentrations of specific chemokines were determined. High CXCL10 levels were detected in acutely infected children regardless of virus pathogen, whereas increased CCL17 production was RSV-specific. Higher percentages of CCR4^+^ CD4 T_EM_ cells in acute RSV infection were accompanied with higher percentages of CXCR3^+^ CD8 T_EM_ cells, whereas the development of long-lived memory CXCR3^+^ CD4 and CD8 T_CM_ cells seems to be compromised, as only children with other viral infections had higher percentages in the convalescent phase. Presence of type-2 and type-1 adaptive antiviral immune response, together with insufficient development of long-lived type-1 T cell memory, could play an important role in RSV pathogenesis and reinfection.

## 1. Introduction

RSV is the most common respiratory pathogen in infants which can cause severe bronchiolitis with pronounced airway hyperreactivity [1]. Frequent rate of RSV reinfections throughout the childhood implicates inadequate development of Th1 and Th2 responses which is evident from simultaneous IFN-*γ* and IL-4/IL-5 production [2, 3].

The efficient antiviral response induces infiltration of Th1 polarized virus-specific CD4 and CD8 T cells via multiple chemokines and their cognate chemokine receptors. Correspondingly, CXCR3 and CCR5 chemokine receptors are expressed on activated type-1 T cells and associated with their local recruitment toward specific chemokines CXCL9, CXCL10, and CCL5 released by RSV-infected epithelial cells [4, 5]. Activated IFN-*γ* producing type-1 T cells are required for the efficient defense against lower respiratory tract RSV infection [6], but overactivated type-1 effector-memory CD8 T cells (T_EM_), observed as the occurrence of high CD8/CD4 T_EM_ cell ratio in lungs [7], can also contribute to the development of more severe pathology and RSV-airway hyperreactivity, respectively. It has also been noticed that, at the peak of the disease, RSV-infected infants have increased CXCL10 levels [8] and enrichment of CXCR3^+^ T cells in lungs that might be responsible for the underlying pathology. CXCR3 expression occurs upon activation on naïve T cells which potently migrate toward cognate CXCL10 ligand [9]. These proinflammatory T cells also coproduce high TNF-*α* levels, which could also be related to the disease severity [10].

RSV has developed different mechanisms to modify a local microenvironment and influence effector-memory differentiation of CD8 T cells [11] irrespective of systemic anti-RSV response [12]. Heidema et al. have shown that lung CD8 T_EM_ cells, known for high IFN-*γ* production and granzyme/perforin-mediated cytotoxicity [7], undergo functional switch to FasL and TRAIL-dependent cytotoxicity that is associated with severe pathology [13].

Another possible underlying mechanism of RSV-mediated interference with antiviral immune response could be the induction of Th2 immune response mediated by thymic stromal lymphopoietin (TSLP), which is capable of modifying Th1-polarization capacity of dendritic cells toward Th2, as observed in infected mice [14]. These dendritic cells release CCL17 and CCL22 chemokines [15, 16] and ultimately potentiate migration of CCR4^+^ Th2 cells [17, 18] involved in type-2 immune response [19]. Moreover, the immune system of newborns favors the Th2 adaptive immune response [2] that might confront efficient Th1 antiviral immune response in primary RSV infection as shown in higher CCR4^+^ to CXCR3^+^ CD4 T cell ratio [20].

In the present study we analyzed systemic adaptive T cell response in the acute and convalescent phase of primary RSV infection in hospitalized infants with bronchiolitis. Increased chemokines CCL17 and CXCL10 levels were detected in serum of infected infants compared to healthy control subjects but increased CCL17 production was strictly RSV-specific. In parallel, higher percentage of CD4 T_EM_ cells expressed CCR4 in the acute RSV phase, with CXCR3 expression comparable to healthy and non-RSV-infected infants.

RSV-infected infants had higher percentages of CD8 T_EM_ cells expressing CXCR3 receptor in the acute infection, but development of long-lived central-memory CXCR3^+^ CD4 and CD8 T cells (T_CM_) seems to be compromised as only infants with other viral infections had higher percentages in the convalescent phase.

## 2. Materials and Methods

### 2.1. Patients

During two respiratory infectious seasons blood samples were collected from children under 2 years of age hospitalized due to severe acute respiratory infections caused by RSV or other viruses (adenovirus and influenza). Samples were acquired within the first 6 days of hospitalization. Patients receiving steroids, or with ongoing allergies, or with immunologic or hematologic disorders were not included. Second blood drawing was performed 4–6 weeks after first sampling (convalescent phase). Blood samples from healthy children younger than 2 years of age, without clinical evidence of allergic, immunologic, or hematologic disorders, infectious diseases, or ongoing corticosteroid therapy at least during the period of 4 weeks prior to blood drawing, were also collected.

Clinical findings like wheezing, oxygen saturation, respiratory frequency, and heart rate together with length of hospitalization, need for O_2_ supplementation, need for mechanic ventilation, and X-ray findings were individually assessed to describe disease severity. Wheezing was determined with inspection of prolonged expiration and with lung auscultation. It was described with 2 variables: the presence of wheezing and number of days that the wheezing was observed. Chest X-ray findings provided us with the information regarding the presence of bronchiolitis or pneumonia. O_2_ saturation was determined by percutaneous method.

Virus detection in infected children was performed in nasopharyngeal secretions using direct immunofluorescence test (light diagnostics) for respiratory viruses (adenovirus, influenza virus, parainfluenza virus, and RSV). Blood and urine culture was performed only if indicated.

Laboratory parameters such as erythrocyte sedimentation rate (ESR), white blood cell count, and C-reactive protein (CRP) [21], together with pharyngeal and nasopharyngeal swabs, were used to exclude bacterial infections.

The study was approved by the medical ethics committees of participating hospitals and parental/guardian consent was given.

### 2.2. Serum and PBMCs

Peripheral blood was collected into blood sample tubes containing either sodium heparin for isolation of peripheral blood mononuclear cells (PBMCs) or clot activator and silicon for serum isolation (BD Vacutainer Blood Collection Tubes, BD). PBMCs were isolated using density gradient on Ficoll-Paque Plus (GE Healthcare Bio-Sciences AB). Frozen PBMCs were stored in liquid nitrogen until further processing. For each subject, serum was separated and stored at −80°C until further analysis.

### 2.3. Detection of Chemokines

Serum chemokine levels of CXCL9 (MIG), CXCL10 (IP-10), CXCL11 (I-TAC), CCL17 (TARC), and CCL22 (MDC) were determined using commercially available ELISA kits (Quantikine immunoassays, R&D Systems Inc.) according to standard protocol procedure provided by manufacturer. Optical density at 450 nm with wavelength correction at 540 nm was measured on Multiscan Spectrum spectrophotometer (Thermo Fisher Scientific).

### 2.4. Cell Surface Staining

Chemokine receptor expression on T cells was analyzed upon immunostaining procedure as follows; after thawing, cells were simultaneously labeled with antihuman monoclonal antibodies for CD3 (Biolegend), CD4 (Biolegend), CD45Ro (eBioscience), CCR7 (R&D Systems), CCR4, and CXCR3 (both BD Pharmingen). Cell samples were acquired on BD^TM^ LSRII flow cytometer and compensation adjustments were done using single stained PBMCs for each fluorescence channel. Multiparametric data analysis was performed using FlowJo v. 7.6.5. (Tree Star Inc.). T cell population was determined as CD3-positive and further distinguished as Th cells (CD3^+^CD4^+^, in text CD4 T cells) and Tc cells (CD3^+^CD4^−^, in text CD8 T cells). Each of T cell subpopulations was gated based on CD45Ro expression. CD45Ro^+^ cells were analyzed for CCR7 and CCR4/CXCR3 chemokine receptor expression to detect percentage of CCR4^+^/CXCR3^+^ effector-memory (CD45Ro^+^ CCR7^−^) or central-memory (CD45Ro^+^CCR7^+^) T cells.

### 2.5. Statistics

Statistical data analysis was performed using Statistica ver. 7 software package (StatSoft Inc.) with Kruskal-Wallis (nonparametric ANOVA) test to compare tested groups (RSV, non-RSV, and healthy controls), while Mann-Whitney *U* test was used to compare patients during acute and convalescent phase of certain viral infection. All data are described as median values (marked on graphs as a line for each group). Spearman rank test was performed for correlation analysis. The *P* value <0.05 was considered statistically significant.

## 3. Results

### 3.1. Collected Samples

Among 46 tested children, there were 17 RSV positive (age: median 3.5 months, range 0.5–14 months; 7 followed up 4–6 weeks later), 11 with other respiratory viral infections (non-RSV infants; age: median 16 months, range 2–22.4 months; 2 infected with influenza virus and 9 infected with adenovirus; 6 followed up 4–6 weeks later), and 18 healthy controls (age: median 10.5 months, range 0.1–22.4 months). All the tested children were Caucasian.

Clinical parameters revealed that 12 patients in RSV group suffered from wheezing that lasted in average for 3 days (range 1–10 days), and in 4 RSV-infected patients family atopy was noticed. RSV-infected children had O_2_ saturation less than 95% (range 78%–94.6%) but only 4 required oxygen supplementation. Respiratory frequency was in average 48 breaths per minute (range 36–80) and heart rate 145 beats per minute (range 134–176). Only one infant needed to be placed in intensive care unit and stayed there for 3-day observation. None of tested children needed intubation or invasive/noninvasive ventilatory support. To describe disease severity of infected infants, severity score was generated as described in Table 1. Complete demographic and clinical data findings are provided in Table 2.

### 3.2. Chemokine Profile

**Figure 1:**
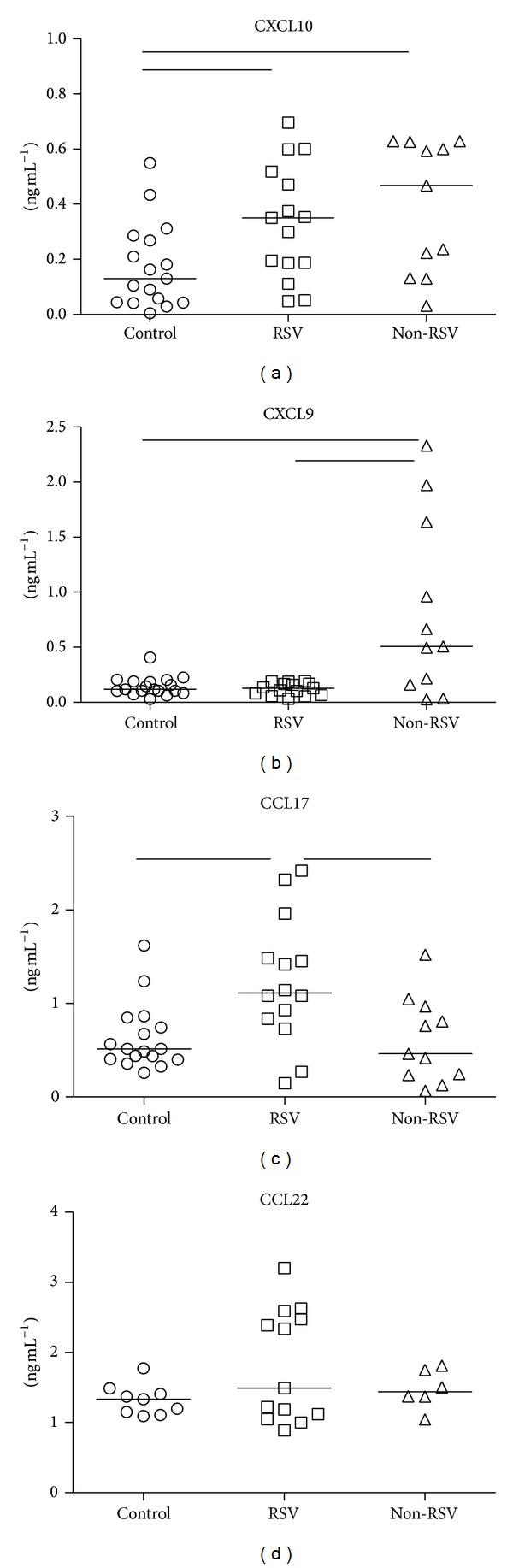
Concentrations of chemokines CXCL10, CXCL9, CCL17, and CCL22 in serum samples. Acutely RSV-infected children (RSV) and children with other respiratory infections (non-RSV) are compared to healthy controls (control). Line in individual groups marks median value. Line between groups presents statistically significant difference (*P* < 0.05).

CXCL9, CXCL10, CXCL11, CCL17, and CCL22 chemokine levels were compared between infected and healthy children. CXCL10 levels (Figure 1(a)) were significantly elevated in acute infections with median concentration 3507 pg/mL for RSV and 4678 pg/mL for other viral infections compared to healthy children (1303 pg/mL). Expression of CXCL9 (Figure 1(b)) was higher in non-RSV children (5081 pg/mL) compared to RSV-infected children (1278 pg/mL) and healthy controls (1172 pg/mL), whereas CXCL11 was not detected in any of tested children (data not shown).

Levels of CCL17 (Figure 1(c)) were significantly elevated (1112 pg/mL) only in RSV-infected children compared to healthy (5129 pg/mL) and non-RSV children (4622 pg/mL). CCL22 serum levels were comparable among healthy and infected children (Figure 1(d)).

### 3.3. Chemokine Receptors CXCR3 and CCR4 on T Cells

**Figure 2:**
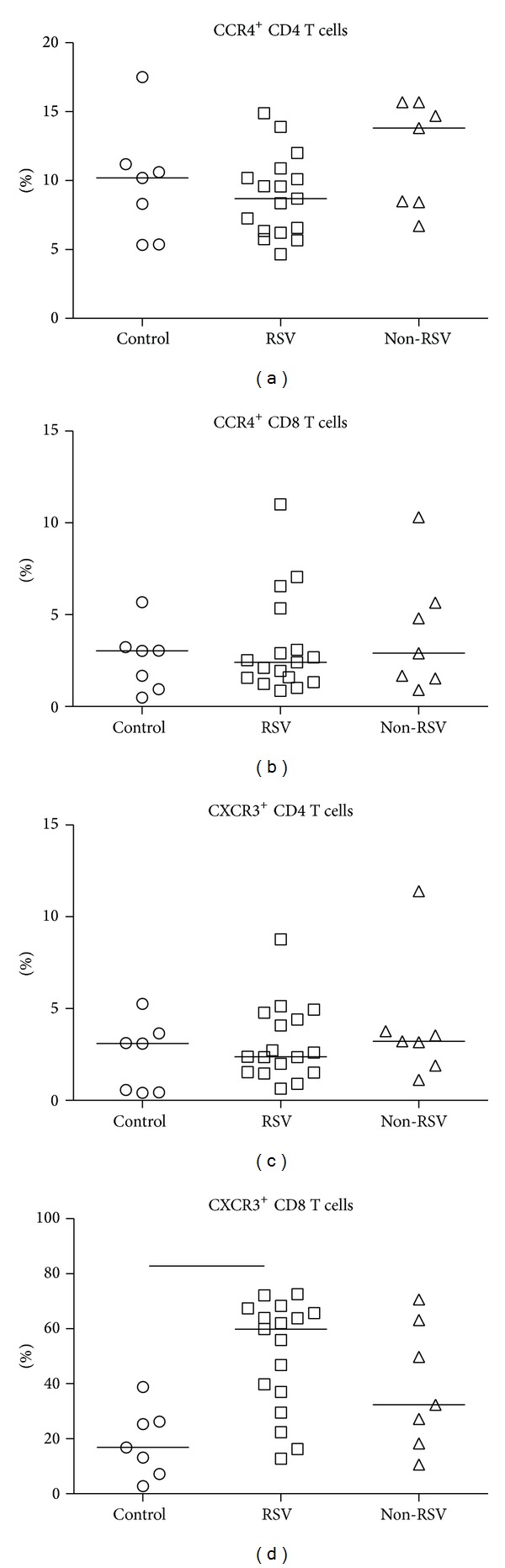
Percentages of chemokine receptors CCR4/CXCR3 on CD4 and CD8 T cells. Acutely RSV-infected children (RSV) and children with other respiratory infections (non-RSV) are compared to healthy controls (control). Line in individual groups marks median value. Line between groups presents statistically significant difference (*P* < 0.05).

Chemokines are responsible for recruitment of lymphocytes expressing specific receptor to the site of infection. Th1-associated CXCL9 and CXCL10 chemokines share specific receptor CXCR3, whereas CCL17 and CCL22 are Th2-associated chemokines which specifically bind to CCR4 receptor. In acute respiratory infections there were no differences in percentages of CXCR3^+^ or CCR4^+^ CD4 T cells compared to healthy children (Figures 2(a) and 2(c)). Expression of CCR4 on CD8 T cells was lower compared to CD4 T cells and did not show statistical difference between tested children (Figure 2(b)). Contrarily, CXCR3^+^ CD8 T cell abundance (Figure 2(d)) in children with acute RSV infections (median 59.9%) was significantly higher compared to non-RSV children (median 32.4%) and healthy controls (median 16.9%).

### 3.4. Chemokine Receptors CCR4 and CXCR3 on Memory T Cells

Cardinal feature of adaptive immunity is fast and efficient response to recall pathogen by antigen experienced memory lymphocytes. Since significant immunopathology in RSV infections coincides with the time of occurrence of antigen specific T cells in lungs and virus frequently causes reinfections, chemokine receptor reflecting Th1 or Th2 immune response was analyzed on effector-memory (CD45Ro^+^CCR7^−^) and central-memory (CD45Ro^+^CCR7^+^) T cells.

**Figure 3:**
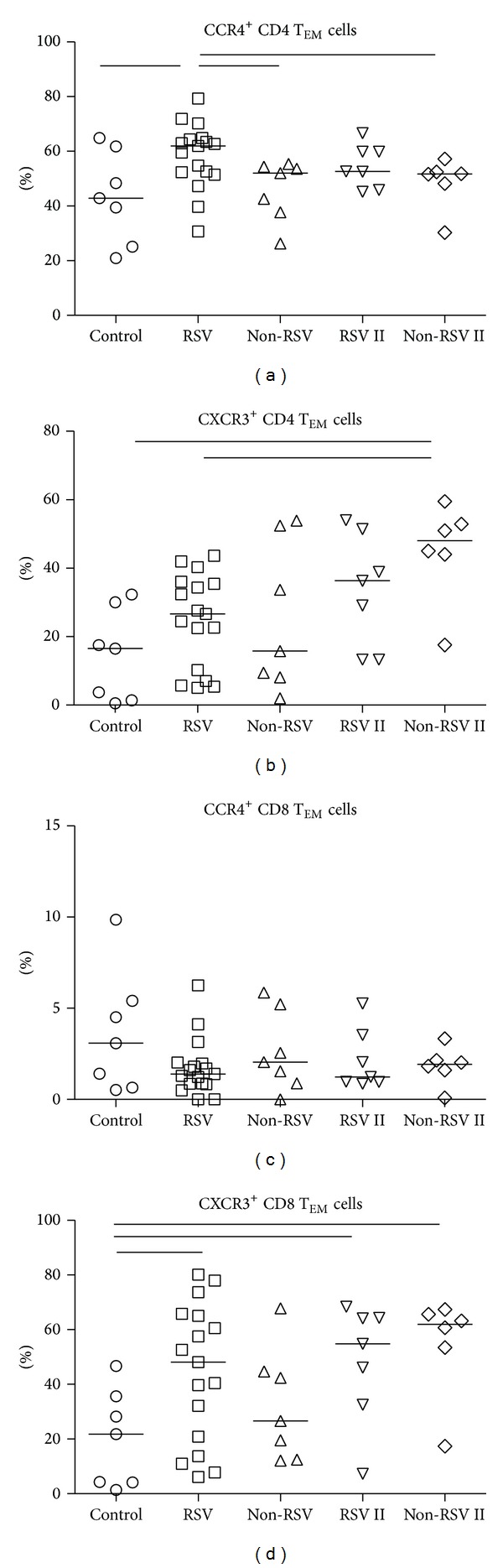
Percentages of chemokine receptor CCR4/CXCR3 on CD4 and CD8 effector-memory T cell (T_EM_). Groups of acutely infected children with RSV (RSV) and with other respiratory infections (non-RSV), children in convalescent phase (RSV II and non-RSV II), and healthy controls (control) are compared using Kruskal-Wallis (nonparametric ANOVA) test. RSV and RSV II or non-RSV and non-RSV II are compared using Mann-Whitney *U* test. Line in individual groups marks median value. Line between tested groups presents statistically significant difference (*P* < 0.05).

Statistically higher percentages of CCR4^+^ CD4 T_EM_ cells (61.94%) distinguished RSV-infected children from other viral infections (52.02%) and healthy controls (42.89%) (Figure 3(a)).

In convalescence, 4–6 weeks after acute infection, the percentage of CCR4^+^ CD4 T_EM_ cells returned to the levels observed for healthy children. Low percentages of CCR4^+^ CD8 T_EM_ (Figure 3(c)) showed no differences between infected children and healthy controls (3.075%), neither in acute (RSV, 1.395%; non-RSV, 2.048%) nor in convalescent phase (RSV, 1.236%; non-RSV, 1.921%).

No differences in expression of CXCR3 on CD4 T_EM_ cells between children with acute respiratory infections and healthy controls (RSV, 26.64%; non-RSV, 15.80%; HC, 16.50%) could be detected. In convalescent phase only infants with other viral infections (48.03%) managed to develop increased percentages of CXCR3^+^CD4 T_EM_ (Figure 3(b)), while children with RSV infection showed only tendency to develop increased percentages of CXCR3^+^CD4 T_EM_ cells.

Percentages of CXCR3^+^ CD8 T_EM_ cells were higher only in RSV-infected children (Figure 3(d)) in acute disease (48.10%) and convalescent phase (54.77%), compared to healthy controls (21.76%). Contrarily, in children with other viral infections percentages of CXCR3^+^ CD8 T_EM_ cells became significantly higher (61.94%) in convalescent phase of infection.

**Figure 4:**
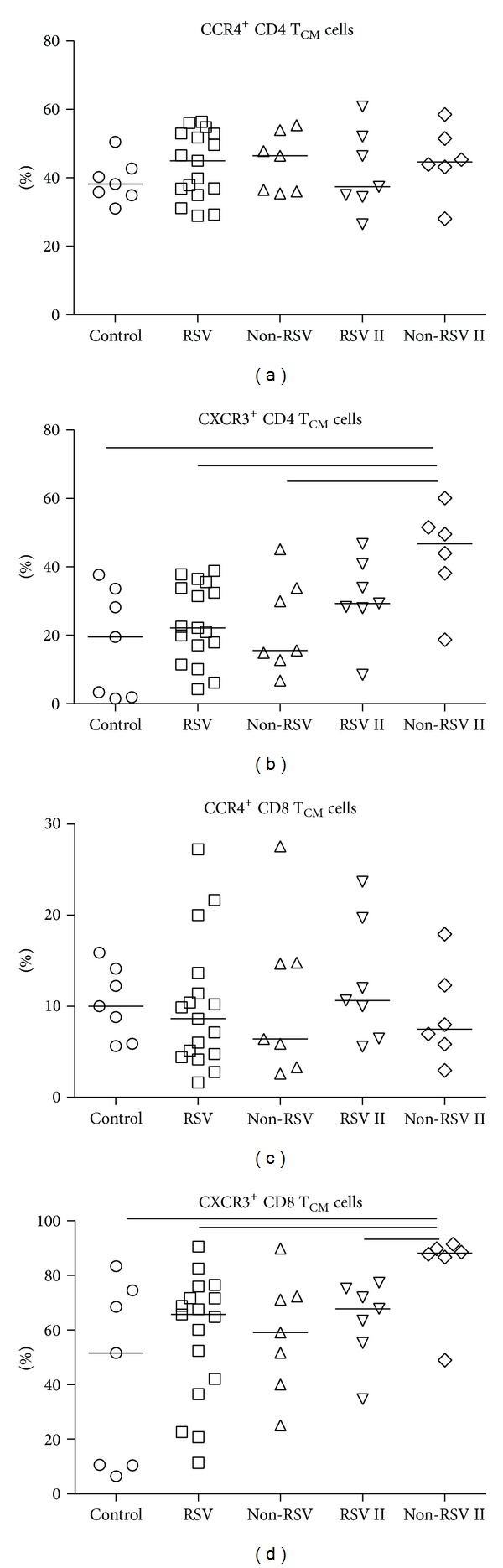
Percentages of chemokine receptor CCR4/CXCR3 on CD4 and CD8 central-memory T cell (T_CM_). Groups of acutely infected children with RSV (RSV) and with other respiratory infections (non-RSV), children in convalescent phase (RSV II and non-RSV II), and healthy controls (control) are compared using Kruskal-Wallis (nonparametric ANOVA) test. RSV and RSV II, or non-RSV and non-RSV II, are compared using Mann-Whitney *U* test. Line in individual groups marks median value. Line between tested groups presents statistically significant difference (*P* < 0.05).

Analysis of CD4 and CD8 T_CM_ cells (Figure 4) in RSV and other respiratory infections revealed no differences in CCR4^+^ receptor expression for either of tested groups, in acute or convalescent phase. On the other hand, children with non-RSV infections showed significant increase in percentages of both CXCR3^+^ CD4 (46.79%) and CD8 T_CM_ cells (67.80%) 4–6 weeks after acute infection (CD4 T_CM_ 15.56%; CD8 T_CM_ 59.10%) compared to healthy controls (CD4 T_CM_ 19.47%; CD8 T_CM_ 51.59%).

### 3.5. Correlations with Disease Severity

Clinical data like wheezing, heart rate, respiratory frequency, and oxygen saturation at admission, together with duration of hospitalization, were collected for infants with acute RSV infection. Higher respiratory frequency, higher heart rate, and lower oxygen saturation related to more severe form of disease. To normalize respiratory frequencies we calculated to which centile the value belongs based on the age of the patient [22]. Centile groups present the deviation from the normally expected respiratory rate values and were numerated and used to correlate with chemokine levels.

**Figure 5:**
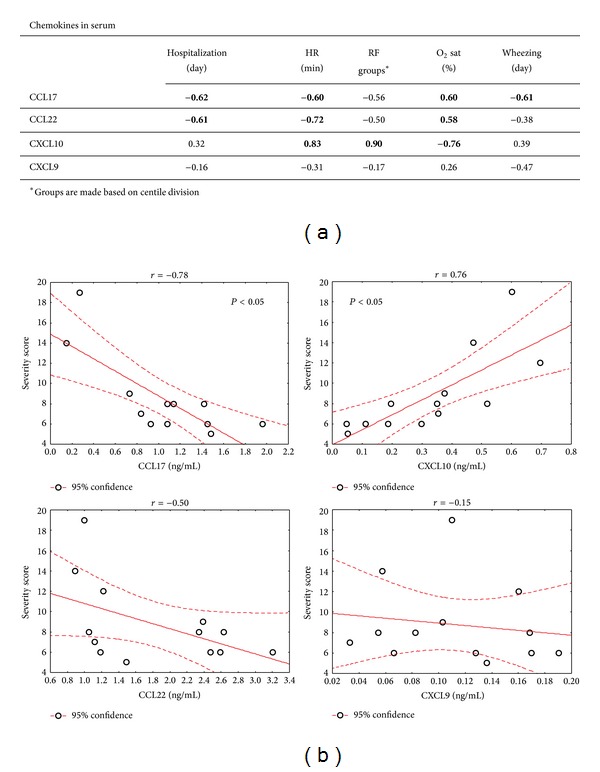
Correlations of chemokine concentrations with clinical data of RSV-infected children. (a) Values in table are Spearman correlation coefficients (*r*). Statistically significant values (*P* < 0.05) are in table marked bold. (b) Graphs present correlation of chemokine with calculated severity score. Statistically significant correlations are marked with *P* < 0.05 sign.

Serum levels of Th2-associated chemokines, CCL17 and CCL22, negatively correlated with disease severity in RSV-infected children (Figure 5(a)), that is, heart rate (*r*_CCL17_ = −0.60; *r*_CCL22_ = −0.72), oxygen saturation (*r*_CCL17_ = 0.60; *r*_CCL22_ = 0.58), and wheezing duration (*r*_CCL17_ = −0.61). On the contrary, serum levels of Th1-associated chemokine CXLC10 levels positively correlated with more severe clinical findings, that is, heart rate (*r* = 0.83), respiratory frequency (*r* = 0.90), and oxygen saturation (*r* = −0.76). Correlations with CXCL10 and CCL17 were confirmed by correlating chemokine levels with disease severity score (Figure 5(b)).

## 4. Discussion

Acute RSV bronchiolitis results in augmented infiltration of activated T lymphocytes at the peak of infection that has been related to the appearance of severe disease as a result of pronounced immunopathology [23]. Since RSV is capable of inducing type-1 and type-2 immune responses simultaneously in infants [2, 19], contribution of each was studied by measuring serum levels of associated chemokines and specific receptor expression on peripheral blood T cells.

Early in life, T cells show intrinsic biased type-2 responses to different pathogens, as Th2 and Th1 cytokines have been detected in nasopharyngeal aspirates from infants infected with different viruses [2]. We detected increased CCL17 levels only in serum of RSV-infected children (Figure 1), which is not surprising since CCL17 is directly induced by infection of respiratory epithelial cells [24] and could be further augmented in the presence of Th2 cytokines [25]. In a similar study, Roe et al. have not found increased CCL17 levels while other chemokine levels were lower compared to our study [20]. This might be due to different sensitivity of the same assay when used for plasma versus serum samples, as indicated by the manufacturer. Moreover, Fujisawa et al. have measured comparable serum CCL17 levels in healthy infants using in-house developed ELISA assay [26]. Additionally, patient cohort recruited by Roe et al. [20] included infants with more severe disease, requiring oxygen supplementation, and different chemokine response could be expected since we found that levels of CCL17 negatively correlate with disease severity (Figure 5).

RSV infection of respiratory epithelial cells induces secretion of both, CCL17 and CCL22, Th2 associated chemokines [24] potentiating local infiltration of CCR4^+^ helper T lymphocytes. Subsequently, Th2 cytokines stimulate epithelial cells to produce excessive levels of CCL17 and CCL22 [25]. Although Th2 immune response confronts efficient anti-RSV response, in a murine RSV-model, IL-4 exerted beneficial effect on the RSV-caused immunopathology by downregulation of inflammatory cytokines [27]. Therefore, it is not surprising that measured serum Th2-related chemokines negatively correlated with disease severity (Figure 5). Many studies emphasized that Th2 cytokines could alter efficient Th1 antiviral immune response and lead to the development of more severe disease, but we have observed a higher IL-4 production in infants with lower respiratory tract infection [19].

The majority of viruses induce CXCL10 expression by the infected cells which is in line with increased CXCL10 serum levels detected in RSV- and other virus-infected children (Figure 1). CXCL10 is the most abundantly present chemokine in the airways of RSV-infected infants [8] originating from infected epithelial cells [5]. High lung CXCL10 levels confirm the development of a Th1 immune response but coincide with exacerbated inflammation involving infiltration of IFN-*γ* producing T cells in murine RSV model [6]. IFN-*γ* levels are inconsistently low in RSV-infected infants and have not shown direct correlation with disease severity [2, 28] but increased and prolonged TNF-*α* production; another Th1 related cytokine has been linked to severe clinical picture in RSV infection. Although CXCL10 is considered a Th1-related chemokine positively regulated by IFN-*γ*, other cytokines like IFN-type I, TNF-*α*, and IL-1*β* also potently induce its production and emphasize CXCL10 as profound proinflammatory chemokine. Therefore, it is not surprising that CXCL10 serum levels positively correlated with aberrant clinical findings (Figure 5). Moreover, blocking CXCL10 receptor decreases airway TNF-*α* mediated hyperresponsiveness and inflammation [29], a common clinical manifestation noticed in severe RSV infection.

Since targeted migration of activated Th1 and Th2 cells via chemokines depends on a specific chemokine receptor expression, we analyzed CCR4 and CXCR3 on peripheral blood T cells. Our results confirmed that percentages of total CCR4^+^ CD4 and CD8 T cells did not differ between study groups, as previously shown by Roe et al. [20], who observed higher CCR4^+^/CXCR3^+^ CD4 T cells ratio.

Additional analysis of CD4 subpopulations revealed an increased percentage of CCR4^+^ CD4 T_EM_ cells only in children with acute RSV infection (Figure 3). The increase was transient since 4–6 weeks later percentages decreased to the levels observed for healthy children. High CCL17 levels accompanied with increased percentage of CCR4^+^ effector-memory Th2 cells during acute RSV infection are probably responsible for the enrichment within lungs and might contribute to the local Th2 cytokine production, confronting efficient antiviral Th1 immune response [30] by increasing activation threshold for Th1 cells and ultimately leading to more severe disease [31, 32].

Similar to CCR4, there was no difference in percentages of total CXCR3^+^ CD4 T cells between infected children at admission and healthy controls (Figure 2). Contrary to CD4 T cells, high percentages of CXCR3^+^ CD8 T cells were acquired in RSV-infected children (Figure 2), confirming their preferential differentiation into type-1 phenotype and importance in antiviral immune responses [33].

As CXCR3 is predominantly expressed on memory T cells, subpopulation analysis revealed increased percentage of peripheral CXCR3^+^ CD8 but no CD4 T_EM_ cells (Figure 3) in acute RSV infection, indicating that augmented antiviral type-1 CTL response is necessary to balance type-2 microenviroment and restrain viral infection [34]. CD8 T_EM_ cells dominate in the lungs of RSV-infected infants and contribute to viral control but could also ultimately exaggerate observed immunopathology [35, 36].

High percentage of CXCR3^+^ CD8 T_EM_ cells in RSV and other virus-infected children remained in periphery beyond clinical disease resolution and might be related to the development of protective short-lived type-1 adaptive immune response. But the development of CXCR3^+^ CD4 and CD8 T_CM_ cells seems to be altered since higher percentages in convalescent phase have been enumerated only in children with other viral infections (Figure 4), addressing possible defect in long-lived type-1 adaptive immune response that could relate to recurrent RSV infections. CXCR3 is early T cell activation marker and designate heterogeneous population in regard to type-1 cytokine production [37]. The majority of these early on activated CXCR3^+^ T cells produce high levels of proinflammatory TNF-*α* but less IFN-*γ*, indicating that additional signals are required to obtain more differentiated polyfunctional T cells. Acute primary RSV infection in infants is accompanied with low IFN-*γ* but high TNF-*α* levels, indicating an incomplete polyfunctional T cell differentiation. Moreover, the predominant TNF-*α* production by type-1 T cells might be related to observed exaggerated inflammation and severe pathology. The possible role for concomitant type-2 response interfering with polyfunctional type-1 response in primary RSV infection should be addressed.

Although a small number of tested patients were included in our study, it confirmed the presence of altered CCR4 and CXCR3 chemokine receptor expression on T cells in children with acute RSV infection. This is related to increased proinflammatory type-1 response and inadequate counterbalance by type-2 response, leading to more severe disease and deregulated development of long-lived memory T cells.

## Figures and Tables

**Table 1:** Disease severity score.

	Score			
	0	1	2	3
Radiological findings and clinical examination	Normal	URTI	Bronchiolitis	Pneumonia
Length of hospitalization (days)	0	1–3	4–10	>10
Duration of stay in intensive care unit	No	N/A	yes	N/A
Wheezing duration (days)	0	1–3	4–7	>7
O_2_ saturation (%)	>95	90–95	80–90	<80
O_2_ supplementation (days)	0	1-2	3-4	>5
Respiratory rate	Normal	N/A	Bradypnea or tachypnea*	N/A
Heart rate	Normal	N/A	Bradycardia or tachycardia*	N/A
Length of fever (days)	0	1-2	2-3	>4

*Based on Pediatric Advanced Life Support (PALS) guidelines for respiratory rate and heart rate.

**Table 2:** Demographic description of tested groups and clinical data of RSV-infected children. For basic statistic data analysis median was used with minimum and maximum range written in parenthesis; *n* marks number; N/A marks nonavailable data.

	RSV	Non-RSV	Controls
*n*	17	11	18
Convalescence (*n*)	7	6	N/A
Age (months)	3.5 (0.5–14)	16 (2–22.4)	10.5 (0.1–22.4)
Gender (% of male)	58.82	36.36	66.66
Family history of atopy (% of subjects)	23.53	9.09	0
Days of symptoms at the time of sample collection	5 (2–10)	4 (2–10)	N/A
Length of hospitalization (day)	5 (1–14)	5 (1–13)	N/A
Wheezing presence (% of subjects)	70.59	0	0
Wheezing duration* (days)	3 (1–10)	N/A	N/A
O_2_ saturation (%)	92 (78–94.6)	>95	N/A
Length of O_2_ supplementation** (days)	5 (2–7)	N/A	N/A
Heart rate (beats/minute)	145 (134–176)	138^+^ (130–160)	N/A
Respiratory frequency (breath/minute)	48 (36–80)	33.5^+^ (24–60)	N/A
White blood cell count (×10^9^/L)	9.6 (5.5–17.1)	19.6 (5.0–36.1)	N/A
CRP (mg/L)	4 (0–44.7)	58.3 (3.4–137)	N/A
Disease severity score	7 (5–19)	5 (3–9)	N/A

*Only for patients with confirmed wheezing.

**Only for patients that needed O_2_ supplementation.

^+^Exact values familiar only for 4 patients, the rest were marked as normal values.
